# Buckwheat anaphylaxis: a case report

**DOI:** 10.1186/1710-1492-10-S2-A38

**Published:** 2014-12-18

**Authors:** Persia Pourshahnazari, Gordon Sussman

**Affiliations:** 1 Department of Internal Medicine, University of British Columbia, Vancouver, British Columbia, Canada; 2 Department of Clinical Immunology and Allergy, University of Toronto, Toronto, Ontario, Canada

## Case

A healthy 32-year-old Chinese female presented with three episodes of allergic reactions after ingestion of buckwheat. She was assessed in the allergy clinic after eating cake containing buckwheat flour. After a few bites she experienced tongue tingling and throat tightness. Within 30 minutes she developed urticaria, nausea and abdominal cramping, which subsided with diphenhydramine. Eight months prior she had an episode of abdominal cramping, urticaria and lip angioedema within 45 minutes of eating multigrain toast. Two years earlier she had eaten buckwheat noodles at a restaurant in China. She developed abdominal cramping, emesis and throat tightness 30 minutes after ingestion, and was treated at a local emergency department. There was no other history of food, drug, insect or latex allergy.

Skin prick testing was performed for food allergies. All food skin tests were negative with appropriate controls. Skin prick testing was performed to the extracted cake and was strongly positive at 9mm (W9F29). Prick testing to extracted buckwheat was remarkably positive at 48mm (W48F70). Specific IgE to buckwheat was obtained and was high at 6.13KU/L.

## Discussion

Common (*Fagopyrum esculentum*) and tartary (*Fagopyrum tartaricum*) buckwheat are members of the Polygonaceae family that are taxonomically unrelated to wheat [1,2]. They contain no gluten and have emerged as a popular substitute for celiac or wheat intolerant patients. Buckwheat has been described as a potent allergen in Asia where it is commonly consumed, with fewer cases described in Europe and North America [1]. Severe symptoms including anaphylaxis can occur after ingestion or inhalation of buckwheat, with Fag e 1 and Fag e 2 proteins identified as major allergens [3].

## Conclusions

Buckwheat represents a major food allergen in Asia where consumption is high. With growing popularity in North American diets, increased awareness is necessary as exposures to this potent allergen become more common.

## Consent

Written informed consent was obtained from the patient for publication of this abstract and any accompanying images. A copy of the written consent is available for review by the Editor of this journal.
